# Supplementary material for: Systemic inflammatory response index is associated with acute kidney injury following cardiac surgery: A retrospective cohort study using the MIMIC database
Source: PLoS One. 2026 Mar 10;21(3):e0342780. doi: 10.1371/journal.pone.0342780 (PMC12974804; doi:10.1371/journal.pone.0342780)
Supplement: Supplementary Material S1 — (DOCX) [file pone.0342780.s001.docx]

**ICD-9 Procedure Codes:** 3512, 3514, 3521, 3522, 3523, 3524, 3527, 3611, 3612, 3613, 3614, 3615, 3845

**ICD-10 Procedure Codes:** 0210093, 0210099, 021009W, 02100A3, 02100A8, 02100A9, 02100AW, 02100Z8, 02100Z9, 0211093, 0211099, 021109W, 02110A9, 02110AW, 02110Z3, 02110Z8, 02110Z9, 0212093, 0212099, 021209W, 02120AW, 02120Z9, 0213093, 0213099, 021309W, 02QF0ZZ, 02QG0ZZ, 02RF08Z, 02RF0JZ, 02RF0KZ, 02RG08Z, 02RG0JZ, 02RG0KZ, 02RJ08Z, 02RW0JZ, 02RX08Z, 02RX0JZ
