# Supplementary material for: Systemic inflammatory response index is associated with acute kidney injury following cardiac surgery: A retrospective cohort study using the MIMIC database
Source: PLoS One. 2026 Mar 10;21(3):e0342780. doi: 10.1371/journal.pone.0342780 (PMC12974804; doi:10.1371/journal.pone.0342780)
Supplement: Attachment — Submitted filename: Response to Reviewer #2.docx [file pone.0342780.s002.docx]

Dear Reviewer #2,

We sincerely appreciate your thorough review and constructive suggestions. Your comments have been extremely helpful in improving the rigor and clarity of our manuscript. Below, we provide a point-by-point response to each of your concerns.

**Comment 1: Resolve the AKI definition (ICD code vs KDIGO labs) and explain the drastic drop in sample size after each inclusion/exclusion step.**

**Response:**

We thank the reviewer for identifying this inconsistency. In our original manuscript, we mistakenly mentioned "ICD-10 code N179" in the Study Design section, which was inconsistent with our actual methodology. AKI was defined according to the KDIGO guidelines based on serum creatinine changes within seven days post-surgery. We have removed the erroneous ICD-10 statement from the Methods section to ensure consistency.

Regarding the sample size reduction, the most substantial drop occurred at the step of excluding patients with insufficient data to calculate SIRI (N=4,212). This is attributable to the significant missing data in the MIMIC database, particularly for differential blood cell counts (neutrophils, monocytes, and lymphocytes) required for SIRI calculation. The MIMIC database, while comprehensive, has inherent limitations in data completeness, especially for laboratory values that may not be routinely ordered for all patients.

**Comment 2: Specify which ICD codes were used to identify cardiac surgery, and justify the exclusion of malignancy.**

**Response:**

We have provided the complete list of ICD-9 and ICD-10 procedure codes used to identify cardiac surgery patients in a new Supplementary Material S1. The Methods section now references this supplementary material:

*"We identified eligible patients using cardiac surgery-related procedure codes from the ICD-9 and ICD-10 (codes are listed in Supplementary Material S1)."*

Regarding the exclusion of patients with malignancy: this decision was based on the fact that malignancy is associated with altered inflammatory status, including abnormal white blood cell counts and differential proportions. Cancer patients often exhibit elevated neutrophil counts and reduced lymphocyte counts due to the disease itself or treatment effects (chemotherapy, radiation). These alterations could significantly confound the relationship between SIRI and AKI, as SIRI is calculated from these cell populations. Therefore, excluding malignancy patients helps ensure that the observed association reflects the inflammatory response related to cardiac surgery rather than cancer-related immune dysregulation.

**Comment 3: Discuss missing important confounders (surgical factors, transfusions, etc.) and consider whether any are available in MIMIC. If possible, perform sensitivity analyses (e.g. stratify by surgical type) or at least acknowledge this limitation explicitly.**

**Response:**

We greatly appreciate this suggestion. We have addressed this concern in two ways:

1. Sensitivity analysis by surgery type:

As suggested, we performed subgroup analysis stratified by surgery type (CABG vs. non-CABG). The results showed that the association between SIRI and AKI was consistent across surgery types (P for interaction = 0.171), with a numerically stronger effect in the non-CABG group (OR: 1.195, 95% CI: 1.044-1.368) compared to the CABG group (OR: 1.064, 95% CI: 0.959-1.180). These results have been added to Table 3 and described in the Results section.

Additionally, we included surgery type as a covariate in Model 4, and the association between SIRI and AKI remained significant after this adjustment.

2. Acknowledgment in Limitations:

We have explicitly acknowledged the unavailability of detailed surgical variables in the Limitations section:

*"Third, due to the limitations of the MIMIC database, detailed intraoperative variables such as cardiopulmonary bypass (CPB) use, CPB duration, aortic cross-clamp time, and blood transfusion were not available for analysis. These factors are known to influence postoperative AKI risk and should be considered in future prospective studies."*

**Comment 4: Correct terminology (use "retrospective cohort" instead of "cross-sectional"), and ensure consistency between text, figure, and table data (the flow chart numbers should match the Methods description).**

**Response:**

We thank the reviewer for this correction. We have revised the study design terminology throughout the manuscript:

Title: Changed from *"A Comprehensive Analysis"* to *"A Retrospective Cohort Study"*

Abstract Methods: Changed from *"retrospective analysis"* to *"retrospective cohort study"*

Methods section: Changed from *"cross-sectional design"* to *"retrospective cohort design"*

Regarding the consistency between text, figure, and table data: we have carefully verified that all numbers in the flow chart (Figure 1) match the descriptions in the Methods section. The sample sizes at each inclusion/exclusion step are consistent throughout the manuscript.

**Comment 5: Strengthen the Introduction/Discussion by citing related studies (e.g. SIRI in AKI prediction, SII after CABG). This will better situate the work in existing research.**

**Response:**

We appreciate this suggestion. We have strengthened both the Introduction and Discussion sections by adding relevant literature on SIRI and SII in AKI prediction:

In Introduction:

*"More recently, SIRI has shown promise in predicting AKI in other clinical settings, such as sepsis and contrast exposure."*

In Discussion:

*"More recently, composite inflammatory indices have attracted increasing attention. The systemic immune-inflammation index (SII) has been shown to be associated with AKI after cardiac surgery in a large cohort study using the MIMIC-IV database (DOI: 10.3389/fcvm.2024.1430776). SIRI has also demonstrated predictive value for AKI in other clinical settings, including acute pancreatitis and contrast-associated AKI following elective PCI. However, the specific relationship between SIRI and cardiac surgery-associated AKI has not been investigated."*

These additions better situate our work within the existing literature and highlight the novelty of our study.

**Comment 6: Temper claims about SIRI being a "predictor" and emphasize that the association is modest (OR ~1.3). Highlight that SIRI may add value to risk stratification, but further validation is needed.**

**Response:**

We completely agree with this comment. We have revised the manuscript throughout to temper our claims. Specifically, we replaced terms like "predictor" and "predicts" with more cautious expressions such as "is associated with" and "risk marker." We also explicitly acknowledged the modest effect size in the Discussion and Conclusion sections, and emphasized that SIRI may serve as a "supplementary risk stratification marker" rather than a definitive predictive tool. Furthermore, we added statements highlighting the need for external validation before clinical application. These revisions are reflected in the Title, Abstract, Discussion, and Conclusion sections.

We believe these revisions have substantially improved the rigor and clarity of our manuscript. We are grateful for your thorough review and constructive suggestions.

Sincerely,

The Authors
